# Cell Proliferation and Neurogenesis in Adult Mouse Brain

**DOI:** 10.1371/journal.pone.0111453

**Published:** 2014-11-06

**Authors:** Olivia L. Bordiuk, Karen Smith, Peter J. Morin, Mikhail V. Semënov

**Affiliations:** New England Geriatric Research Education and Clinical Center, Bedford Division, Edith Nourse Rogers Memorial Veterans Hospital, Bedford, Massachusetts, United States of America; Central Michigan University, United States of America

## Abstract

Neurogenesis, the formation of new neurons, can be observed in the adult brain of many mammalian species, including humans. Despite significant progress in our understanding of adult neurogenesis, we are still missing data about the extent and location of production of neural precursors in the adult mammalian brain. We used 5-ethynyl-2'-deoxyuridine (EdU) to map the location of proliferating cells throughout the entire adult mouse brain and found that neurogenesis occurs at two locations in the mouse brain. The larger one we define as the main proliferative zone (MPZ), and the smaller one corresponds to the subgranular zone of the hippocampus. The MPZ can be divided into three parts. The caudate migratory stream (CMS) occupies the middle part of the MPZ. The cable of proliferating cells emanating from the most anterior part of the CMS toward the olfactory bulbs forms the rostral migratory stream. The thin layer of proliferating cells extending posteriorly from the CMS forms the midlayer. We have not found any additional aggregations of proliferating cells in the adult mouse brain that could suggest the existence of other major neurogenic zones in the adult mouse brain.

## Introduction

Neurogenesis, the formation of new neurons, can be observed in the adult brain of many mammalian species including humans. In the hippocampus, the new neurons are incorporated into the dentate gyrus and contribute to neuronal plasticity, particularly to the formation of new memories and learning [1]–[3]. Another location for neurogenesis is the olfactory bulbs, where new neurons are incorporated to replace worn out olfactory interneurons [4]–[6]. There are also reports of new neuron incorporation in other parts of the adult brain. However, their origin, role, and extent of incorporation is still not fully characterized [6]–[8].

Age, trauma, and neurodegenerative diseases all lead to the loss of cognitive, motor, and analytical potency in the brain. This decline is in part attributed to the loss of neurons. Studying adult neurogenesis should help us understand how we may use endogenous adult-born neurons for brain repair and restoration. Despite significant progress in our understanding of adult neurogenesis, the extent and location of production of neural precursors in the entire mammalian brain has not been fully characterized. Recently, the thymidine analog 5-ethynyl-2'-deoxyuridine (EdU) was introduced as a tool for robust and simple detection of proliferating cells [9], [10]. We used EdU to locate proliferating cells involved in neurogenesis in the adult mouse brain.

## Results

### Use of EdU for labeling of proliferating cells

**Figure 1:**
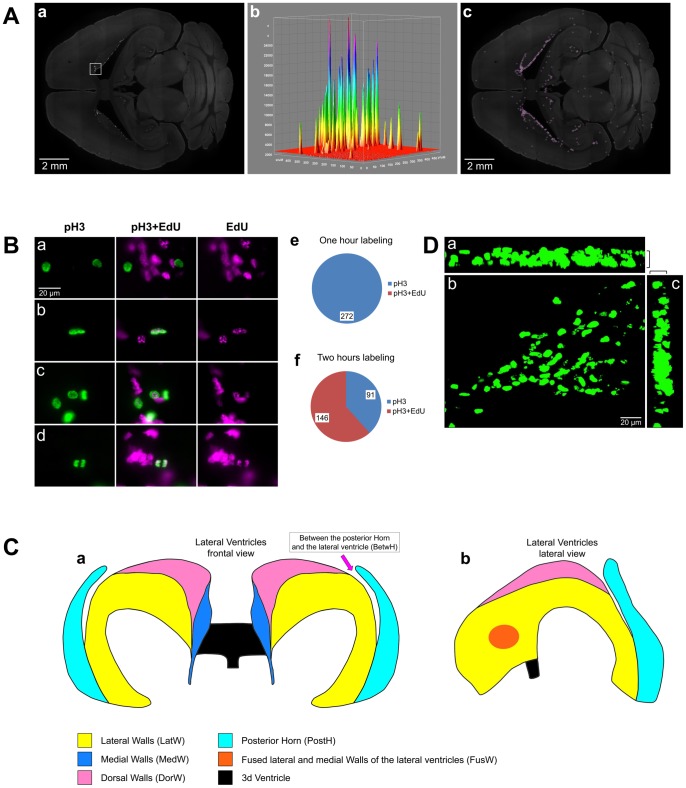
EdU staining. A. EdU staining has a low and homogeneous background. a. EdU staining of a transverse brain section in a 120 day-old mouse one hour post EdU injection. b. 3D surface plot of the area is outlined with a white box on panel (a). c. EdU positive nuclei identified with Find Maxima Process (Fiji image processing package). Each identified nucleus is shown as a magenta dot surrounded by a white crosshair. B. 60% of dividing cells become labeled with EdU two hours after EdU injection. A section of the brain of a 120 day-old mouse stained for phospho-Histone H3 to identify mitotic cells (pH3), EdU (EdU) and phospho-Histone H3 and EdU (pH3+EdU) double stained. a. One hour after EdU injection. b–d. Two hours after EdU injection. Panel b shows a cell at the prophase stage of cell cycle; panel c shows two cells at the metaphase stage; and d a cell at the anaphase stage. e, f. Pie charts showing the number of nuclei stained positive for phospho-Histone H3 alone (blue) and phospho-Histone H3 and EdU (red) ((e) one hour and (f) two hours after EdU injection). C. Structure of the lateral ventricles of the brain of a 120 day-old mouse. The structure was reconstructed using serial transverse sections of the mouse brain, (a) Frontal and (b) lateral view. D. EdU-labeled nuclei are stained through the entire thickness of brain sections. Maximum intensity projections of a 50 µm transverse section of the brain stained for EdU. A point of the rostral migratory stream departure for the anterior end of the caudoputamen is shown. a, Projection along X axis; b, along Z axis; and c, along Y axis. Section thickness along Z axis are shown with brackets. 120 days-old mouse one hour post EdU injection.

We use EdU to label proliferating cells. EdU is a thymidine analog that is incorporated into replicated chromosomal DNA during the S phase of the cell cycle. Detection of incorporated EdU is a simple and robust procedure [9], [10] that allows consistent processing of a large number of mouse brain sections. EdU staining produces a low and homogeneous background that allows us to automatically detect EdU positive nuclei by using the “Find Maxima” process in the Fiji image analysis package (Figure 1A). In addition, EdU labeled nuclei can be stained throughout the entire thickness of the brain sections (Figure 1D) allowing detection of all labeled nuclei. Previous studies using Bromodeoxyuridine (BrdU), another thymidine analog, showed that mitotic cells labeled with BrdU could be observed in adult mouse brain two hours after BrdU injection, and therefore the two hour time point was proposed as an appropriate time for "a true measure of proliferation" [11]. However, we found that two hours after EdU injection about 60% of dividing cells become labeled with EdU and some of them had already proceeded to late anaphase stage with fully separated chromosomes (Figure 1B). Each group of chromosomes was detected as an EdU labeled nucleus in our assay and resulted in double counting of proliferating cells in the late anaphase. In contrast, one hour after EdU injection we did not observe any mitotic cells labeled with EdU showing that one hour is not enough time for the cells to transition from S phase to M phase in the adult brain (Figure 1B). Therefore we use a one hour labeling time in our study instead of the widely accepted two hour labeling time.

### Distribution of proliferating cells in the entire brain of adult mouse

**Figure 2:**
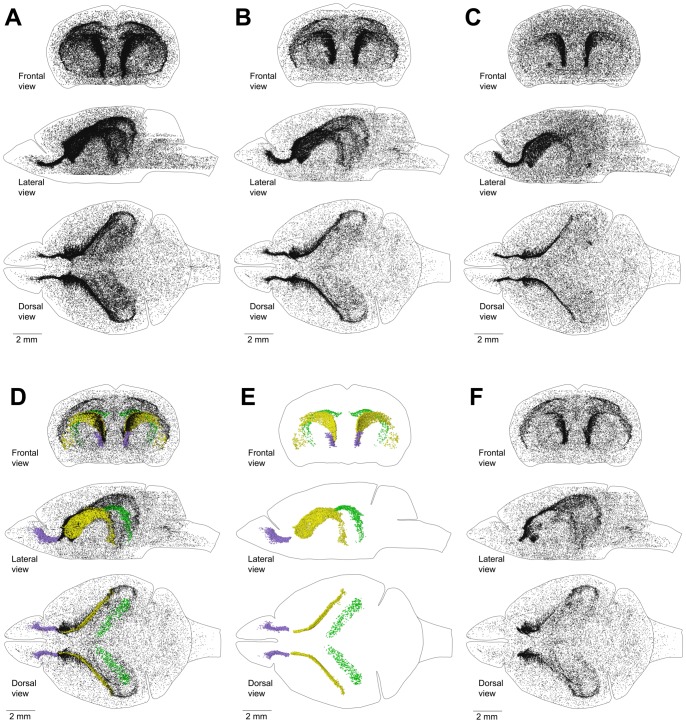
Distribution of proliferating cells in a mouse brain. A–C. Distribution of proliferating cells in the brain of a 60 day-old mouse (A), 120 day-old mouse (B), and 240 day-old mouse (C). Each EdU-labeled nucleus is shown as a black dot. D–F. Distribution of proliferating cells in the lateral walls of the lateral ventricles (LatW), the dentate gyrus of the hippocampus (DG) and the rostral migratory stream (RMS) of a 120 day-old mouse. Each EdU-labelled nucleus is shown as a yellow dot in the LatW, a green dot in the DG, and a lilac dot in the RMS. EdU-labelled nuclei outside of these three structures are shown as black dots of smaller size. D. All proliferating cells in the brain. E. Proliferating cells in the LatW, DG and RMS. F. Proliferating cells located outside the LatW, DG and RMS. The brain shape is outlined with a black line. Frontal, lateral, and dorsal views of the brain are shown. Mice are labeled with EdU for one hour.

Mice are considered to be adults at the age of two months (NIH report, “Animal models”). To ensure that all developmental and adolescence processes were completed in the mouse brain, we used the brain of a four month-old mouse to study the distribution of proliferating cells. To visualize the distribution of proliferating cells in the entire mouse brain, we labeled proliferating cells with EdU for one hour, cut the entire brain transversely in 50 µm sections, stained them for EdU, and obtained images for all sections. Next, we arranged the images in the correct order and orientation, manually registered them, and obtained coordinates for EdU-labeled nuclei on each section. We then combined the coordinates of the EdU-labeled nuclei from all sections and visualized the distribution of proliferating cells in the entire brain as a point cloud. We found that proliferating cells are distributed throughout the entire brain (Figure 2B) with clearly distinguishable cell aggregations in the middle of the left and right hemisphere. The cell density in the aggregations increases progressively from the back of the brain to the front, where the aggregations collapse into narrow bands that dissipate at the entrance into the olfactory bulbs (OB) (Figure 2B). To assess how the distribution of proliferating cells changes with age, we reconstructed the distribution of proliferating cells in the brain of a 60 day-old mouse and a 240 day-old mouse. A sixty day-old mouse is on the verge of adulthood and a 240 day-old mouse is fully mature. We found that the distribution of proliferating cells in these mice is essentially the same as in 120 day-old mice (Figure 2A, C). Despite the similarity in the distribution we found that the number of proliferating cells in the mouse brain decreases with the increase in age (Table 1). In addition, we analyzed another brain of a 120 day-old mouse. The number of proliferating cells that we detected in this brain differs by less than 5% from the cell number we found in the first 120 day-old mouse brain (Table 1). This shows good reproducibility of our approach to the identification of proliferating cells in the mouse brain.

**Table 1:** Number of proliferating cells in the brain of adult male mice.

Mouse	Age	Number of EdU labeled nuclei
60D	60 days-old	68202
120D-A	120 days-old	48574
120D-B	120 days-old	50719
240D	240 days-old	42423

### Main proliferative zone (MPZ)

The lateral walls of the lateral ventricles (LatWs) (Figure 1C) and the dentate gyrus (DG) of the hippocampus are two established places where adult neurogenesis is observed in the mouse brain [6]. We analyzed images of brain sections and manually selected EdU-labeled nuclei located in the LatW, DG and the rostral migratory stream (RMS). The coordinates of these nuclei were then obtained and the distribution of selected cells was visualized as a point cloud with different colors assigned to cells in each structure (Table 2). We also obtained the coordinates of the EdU-labeled nuclei that lay outside of these structures and visualized them as a black dots of smaller size (Figure 2D).The cells in the LatW are colocalized with the middle part of the aggregation of proliferating cells. The cells in the DG are colocalized with a band of proliferating cells in the posterior part of the aggregation, and the cells in the RMS are colocalized with a dense band of proliferating cells at the anterior end of the aggregation. However, the aggregation clearly extends beyond these structures (Figure 2E, F).

**Table 2:** Distribution of proliferating cells in the brain of a 120 day-old male 120D-A mouse.

	Location of proliferating cells	Number of proliferating cells	In MPZ				In DG	Outside of MPZ and DG	Color
			In RMS	In CMS	In Midlayer	Total in MPZ			
BetwH	Between the posterior Horn and the lateral ventricles	435		305	130	435			magenta
Caud	The dorsal and lateral Caudate walls	7900		7900		7900			blue
DG	The Dentate Gyrus	1505					1505		green
DorW	The Dorsal Wall of the lateral ventricles	1154		67	805	872		282	pink
FusW	Fused lateral and medial Walls of the lateral ventricles	1041		1041		1041			orange
LatW	The Lateral Wall of the lateral ventricle	11153		11153		11153			yellow
MedW	The Medial Wall of the lateral ventricle	1231		1110		1110		121	light blue
OB	The Olfactory bulbs	96						96	olive
Other	Other proliferating cells	15543						15543	black
PostH	The lateral wall of the Posterior Horn	506			506	506			cyan
RMS	The Rostral Migratory Stream	3485	3485			3485			lilac
SupAl	Supra Alveus Cells	4525			4525	4525			red
	Total number of proliferating cells	48574	3485	21576	5966	31027	1505	16042	

Main Proliferative Zone (MPZ), Caudate Migratory Stream (CMS).

**Figure 3:**
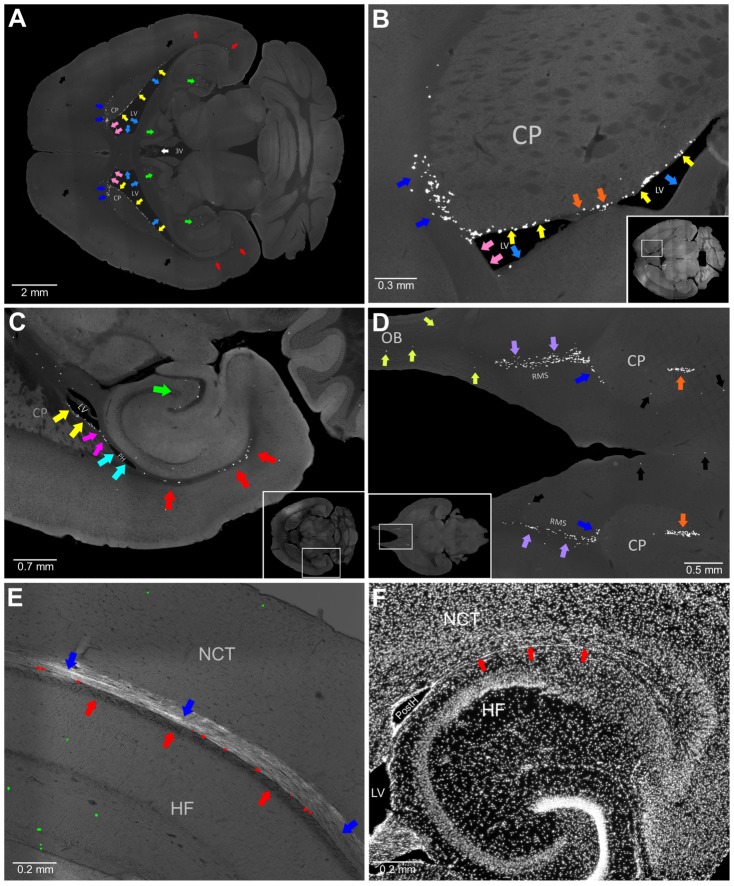
Brain structures appear to be enriched with an abundance of proliferating cells. A. The apparent abundance of proliferating cells is in the supra alveus (SupAl) (red arrows), the dentate gyrus (DG) (green arrows), the lateral walls of the lateral ventricles (LatW) (yellow arrows), the dorsal walls of the lateral ventricles (DorW) (pink arrows), the medial walls of the lateral ventricles (MedW) (light blue arrows), the dorsal and anterior walls of the caudate (Caud) (blue arrows), the choroid plexus in the third ventricle (white arrow), and other parts of the brain (black arrows). B. The apparent abundance of proliferating cells is in the LatW (yellow arrows), the DorW (pink arrows), the MedW (light blue arrows), the Caud (blue arrows), and at the fusion of the lateral and medial walls of the lateral ventricles (FusW) (orange arrows). C. The apparent abundance of proliferating cells is in the SupAl (red arrows), the DG (green arrow), between the posterior Horn and the lateral ventricles (BetH) (magenta arrows), and the lateral wall of the Posterior Horn (PostH) (cyan arrows). D. The apparent abundance of proliferating cells is in the olfactory bulbs (OB) (olive arrows), the rostral migratory stream (RMS) (lilac arrows), the Caud (blue arrows), and the FusW (orange arrows). E. Proliferating cells in the SupAl. EdU-labeled nuclei in the dorsal area of the SupAl are shown as red dots. Other EdU-labeled nuclei are shown as green dots. The image of EdU-labeled nuclei is superimposed on the DIC image of the brain section. The white matter bundles forming the alveus are shown with red arrows and the white matter bundles connecting the thalamus with the cortex with blue arrows. F. A transverse section of the mouse brain stained with Hoechst. The midlayer, identified with red arrows, is a thin sheet of densely packed cells. 3rd ventricle (3V), Caudoputamen (CP), Hippocampal Formation (HF), Lateral Ventricles (LV), Neocortex (NCT), Olfactory Bulbs (OB), Posterior Horn (PostH). Rostral Migratory Stream (RMS). 120 days-old mouse one hour post EdU injection.

**Figure 4:**
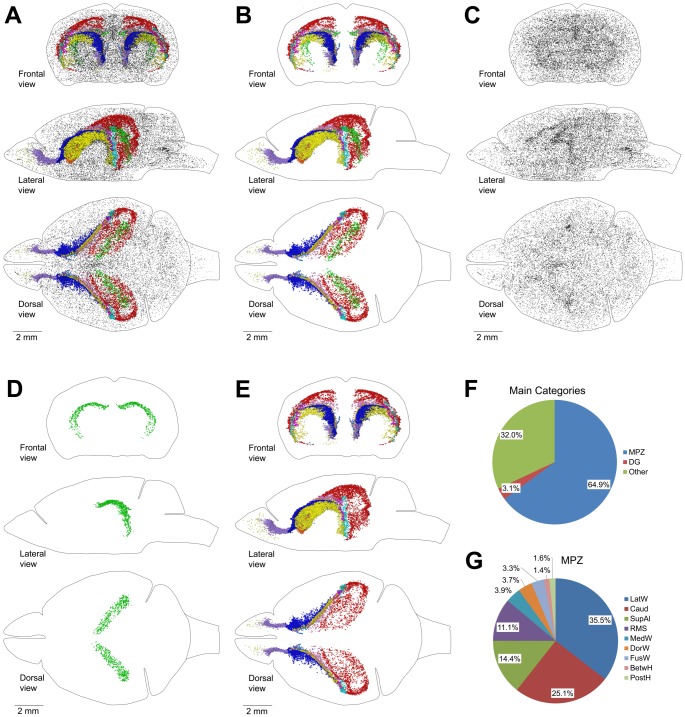
Main Proliferative Zone (MPZ). A–E. Distribution of proliferating cells in the brain of a 120 day-old mouse labeled with EdU after one hour. Each EdU-labeled nucleus is shown as a colored dot with colors assigned according to the Table 2. The brain shape is outlined with a black line. Frontal, lateral, and dorsal views of the brain are shown. A. Distribution of EdU-labeled nuclei in the brain. B. Distribution of EdU-labeled nuclei in the aggregation. C. Distribution of EdU-labeled nuclei outside of the aggregation. D. Distribution of EdU-labeled nuclei in the dentate gyrus (DG). E. Distribution of EdU-labeled nuclei in the MPZ. F. Pie chart showing the percentage of EdU labeled nuclei located in the MPZ, DG and other parts of the adult mouse brain (Other). G. Pie chart showing the percentage of EdU labeled nuclei located in different parts of the MPZ. Abbreviated names are given according to Table 2.

**Figure 5:**
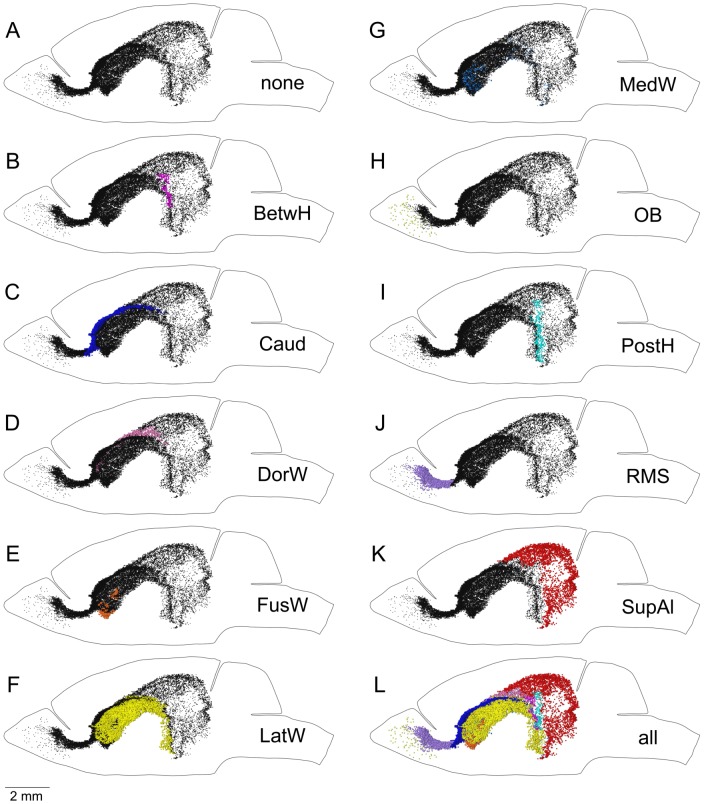
The Main Proliferative Zone (MPZ) appears as an uninterrupted continuum in which EdU-labeled nuclei from each structure occupy well-defined locations. A. Each EdU-labeled nucleus in the MPZ is shown as a black dot. B. Each EdU-labeled nucleus in the BetwH is shown as a magenta dot. EdU-labeled nuclei in other parts of the MPZ are shown as black dots. C. Each EdU-labeled nucleus in the Caud is shown as a blue dot. EdU-labeled nuclei in other parts of the MPZ are shown as black dots. D. Each EdU-labeled nucleus in the DorW is shown as a pink dot. EdU-labeled nuclei in other parts of the MPZ are shown as black dots. E. Each EdU-labeled nucleus in the FusW is shown as an orange dot. EdU-labeled nuclei in other parts of the MPZ are shown as black dots. F. Each EdU-labeled nucleus in the LatW is shown as a yellow dot. EdU-labeled nuclei in other parts of the MPZ are shown as black dots. G. Each EdU-labeled nucleus in the MedW is shown as a light blue dot. EdU-labeled nuclei in other parts of the MPZ are shown as black dots. H. Each EdU-labeled nucleus in the OB is shown as an olive dot. EdU-labeled nuclei in other parts of the MPZ are shown as black dots. I. Each EdU-labeled nucleus in the PostH is shown as a cyan dot. EdU-labeled nuclei in other parts of the MPZ are shown as black dots. J. Each EdU-labeled nucleus in the RMS is shown as a lilac dot. EdU-labeled nuclei in other parts of the MPZ are shown as black dots. K. Each EdU-labeled nucleus in the SupAl is shown as a red dot. EdU-labeled nuclei in other parts of the MPZ are shown as black dots. L. Each EdU-labeled nucleus in the MPZ is shown as a color dot with colors assigned according to the Table 2. Abbreviated names are given according to Table 2. The brain shape is outlined with a black line. Lateral view. 120 days-old mouse one hour post EdU injection.

**Figure 6:**
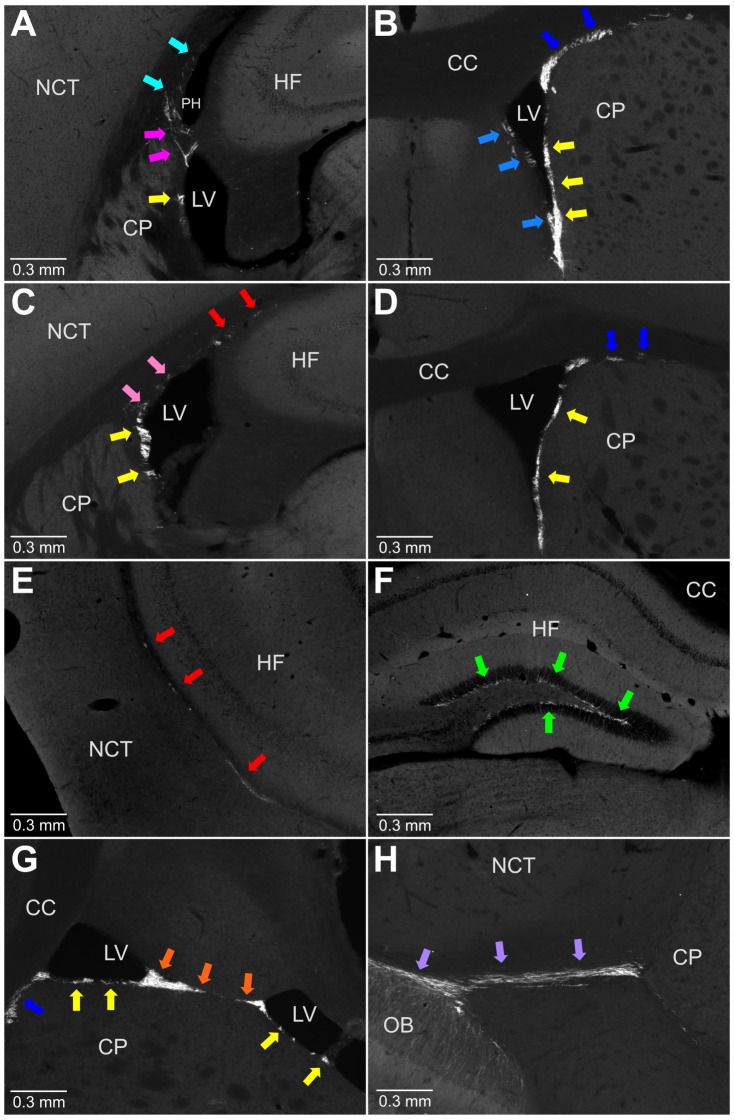
Doublecortin expressing cells are present in all parts of the main proliferative zone. A-F, coronal sections and G, H, transverse sections. A. Presence of doublecortin expressing cells in the LatW (yellow arrow), BetH (magenta arrows), and PostH (cyan arrows). B. Presence of doublecortin expressing cells in the LatW (yellow arrows), MedW (light blue arrows), and Caud (blue arrows). C. Presence of doublecortin expressing cells in the LatW (yellow arrows), DorW (pink arrows), and SupAl (red arrows). D. Presence of doublecortin expressing cells in the LatW (yellow arrows) and Caud (blue arrows). E. Presence of doublecortin expressing cells in the SupAl (red arrows). F. Presence of doublecortin expressing cells in the DG (green arrow). G. Presence of doublecortin expressing cells in the LatW (yellow arrows), FusW (orange arrows), and Caud (blue arrow). H. Presence of doublecortin expressing cells in the RMS (lilac arrows). Corpus Callosum (CC), Caudoputamen (CP), Hippocampal Formation (HF), Lateral Ventricles (LV), Neocortex (NCT), Olfactory Bulbs (OB), Posterior Horn of the lateral ventricles (PH). Abbreviated names are given according to Table 2. 120 day-old mouse.

We examined brain sections and selected eight additional brain structures that appeared to be enriched with an abundance of proliferating cells (Table 2, Figure 3A–D). Next, we obtained coordinates of EdU-labeled nuclei in each of these structures and visualized their distribution inside the mouse brain as a point cloud with different colors assigned to cells from each structure (Table 2, Figure 4A). The point cloud of all selected cells (Figure 4B) closely resembled the aggregation (Figure 2B). Unselected EdU-labeled nuclei showed no additional aggregations (Figure 4C). EdU-labeled nuclei in the aggregation formed two separate point clouds. The cells distributed along the DG formed one cloud (Figure 4D), and the cells in the ten other structures formed another that we named the Main Proliferative Zone (MPZ). The MPZ appears as an uninterrupted continuum (Figure 4E) in which EdU-labeled nuclei from each structure occupy well-defined locations (Figure 5). We also found that cells expressing doublecortin (DCX) are present in all structures comprising the MPZ (Figure 6).

**Figure 7:**
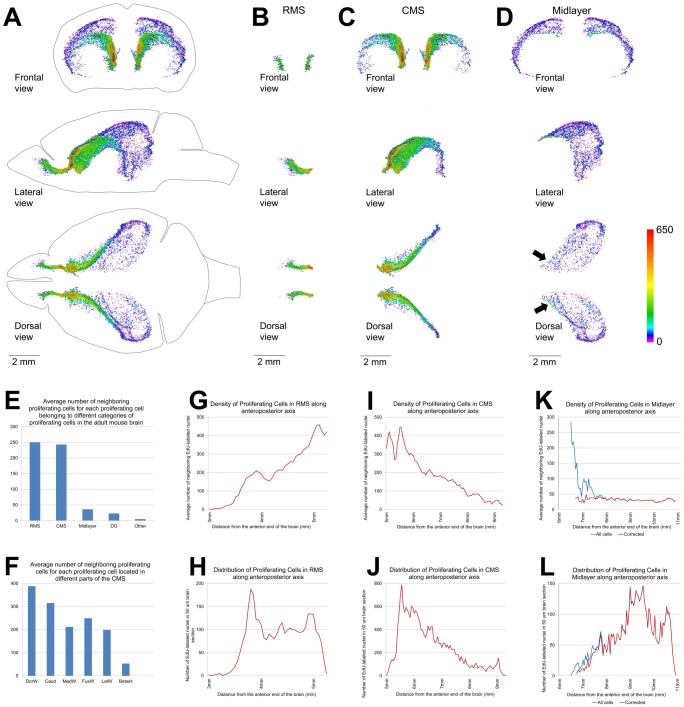
The density of proliferating cells in the Main Proliferative Zone (MPZ). A–D. Volume number density of proliferating cells in the MPZ (A), the rostral migratory stream (RMS)(B), the caudate migratory stream (CMS) (C), the midlayer (D). Each EdU-labeled nucleus is shown as a colored dot with colors assigned according to the number of neighboring EdU-labeled nuclei located closer than 200 µm to this nucleus. Scale is shown on the right. E–F. Average number of neighboring EdU-labeled nuclei located closer than 200 µm to each EdU-labeled nucleus from main categories of proliferating cells in the adult mouse brain (E) or different parts of the caudate migratory stream (F). Abbreviated names are given according to Table 2. G, I, K. Density of proliferating cells in the RMS (G), CMS (I) and midlayer (K) along the anteroposterior axis of the brain. The average number of neighboring EdU-labeled nuclei located closer than 200 µm to each EdU-labeled nucleus in a 50 µm brain section is shown along the vertical axis of the charts. The distance from the anterior end of the brain is shown along the horizontal axis of the charts. H, J, L. Distribution of proliferating cells in the RMS (G), CMS (I) and midlayer (K) along the anteroposterior axis of the brain. The number of EdU-labeled nuclei in a 50 µm brain section is shown along the vertical axis of the charts. The distance from the anterior end of the brain is shown along the horizontal axis of the charts. K, L. The blue line shows the density (K) and distribution (L) of all proliferating cells located in the midlayer. The red line shows the density (K) and distribution (L) of proliferating cells located in the midlayer, with the exception of 250 cells located close to the CMS (these cells are shown with black arrow on panel D). 120 days-old mouse one hour post EdU injection.

About two thirds of all proliferating cells in the adult mouse brain are located in the MPZ (Figure 4F). The number of proliferating cells located in the DG is about twenty times less. The remaining third are located outside of the aggregation (Figure 4C, F, G). The volume number density of proliferating cells in the MPZ shows that the cells have a high density in the anterior and medial parts and a much lower density in the posterior part (Figure 7A) suggesting that the MPZ may be comprised of a number of sub regions.

### Rostral Migratory Stream (RMS)

**Figure 8:**
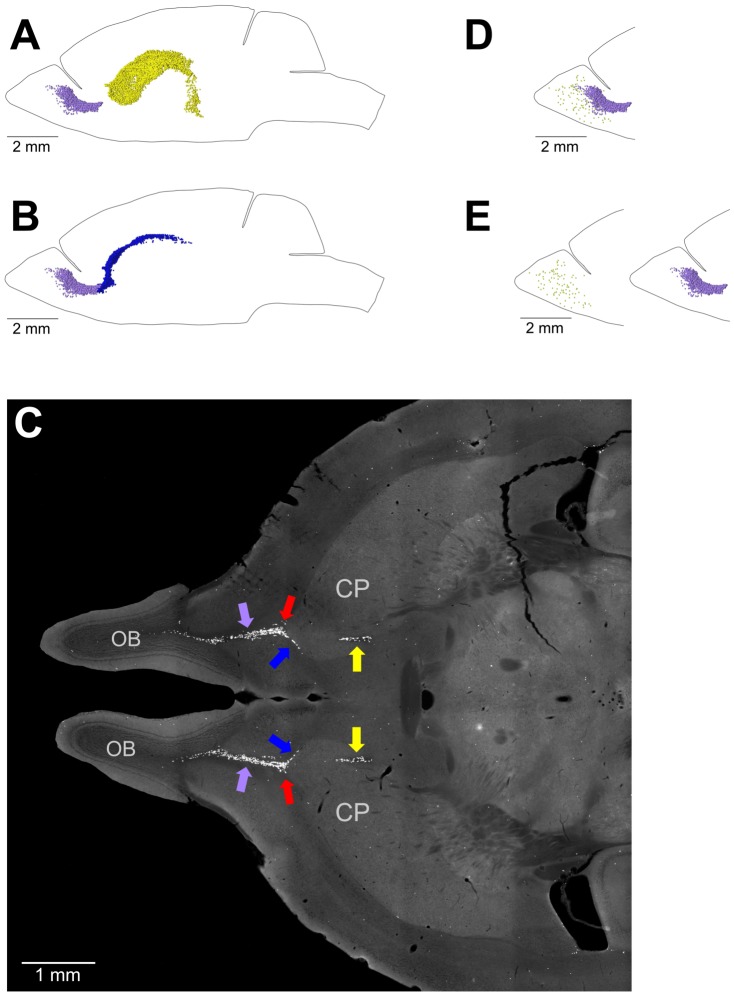
Transition between the Caudate Migratory Stream (CMS) and Rostral Migratory Stream (RMS) and the RMS and olfactory bulbs (OB). A–C. Transition between the CMS and RMS. The RMS in the adult mouse brain departs from the most anterior end of the caudoputamen (CP). A. The distribution of EdU-labeled nuclei in the lateral walls of the lateral ventricles (shown as yellow dots) has no overlap with the distribution of EdU-labeled nuclei in the RMS (shown as lilac dots). B. The distribution of EdU-labeled nuclei in the dorsal and lateral caudate walls (shown as blue dots) overlaps with the distribution of EdU-labeled nuclei in the RMS (shown as lilac dots). C. Transverse section of the mouse brain stained for EdU. EdU-labeled nuclei in the caudate walls are shown with blue arrows, in the RMS with lilac arrows, in the lateral walls of the lateral ventricles with yellow arrows and the point of transition between the caudate walls and the RMS with red arrows. D, E. Transition between the RMS and OB. The stream of proliferating cells in the RMS does not extend into the olfactory bulbs. EdU-labeled nuclei in the RMS are shown as lilac dots and in the OB as olive dots. EdU-labeled nuclei in the RMS and OB are shown as they are located in the mouse brain (D) or virtually separated by 4 mm to show that the stream of proliferating cells in the RMS does not extend into the OB (E). 120 days-old mouse one hour post EdU injection.

The most anterior part of the MPZ is the RMS. This part of the MPZ can be readily identified as a cable of proliferating cells emanating from the most anterior part of the caudoputamen toward the OB (Figure 8A–C; 3D; 9Bd). The RMS contains about 11% of the proliferating cells in the MPZ (Figure 10I). Proliferating cells have a very high volume number density near the caudoputamen that progressively decreases toward the anterior end of the RMS (Figure 7B, G). At same time, the number of proliferating cells in the RMS remains fairly constant along the anteroposterior axis (Figure 7H) showing that the cells become less tightly packed toward the anterior end of the RMS. We have not noticed any aggregation of proliferating cells in the OB that could be a continuation of the RMS (Figure 8D, E). Proliferating cells in the OB are distributed rather sparsely, similar to the distribution in other parts of the brain outside of the aggregation (Figure 11A). Therefore, we concluded that the RMS is not extending into the OB.

**Figure 9:**
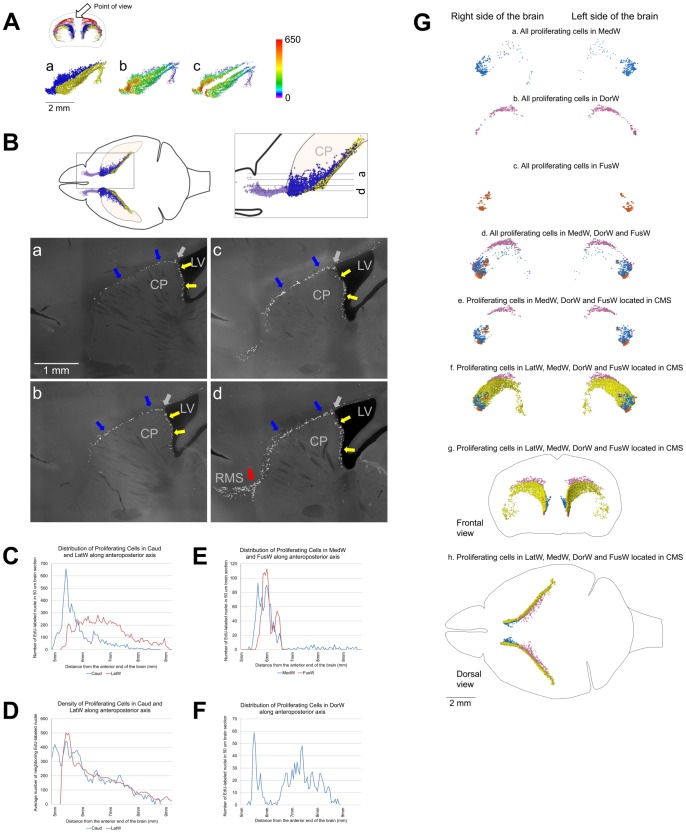
Caudate Migratory Stream (CMS). A–D. Proliferating cells in the medial wall of the caudate facing the lateral ventricles and in the dorsal and lateral walls have similar distribution. A. Distribution of proliferating cells in the lateral walls of the lateral ventricles (LatW) formed by the medial wall of the caudate and in the dorsal and lateral walls of the caudate. a. EdU-labeled nuclei in the LatW are shown as yellow dots and in the dorsal and lateral walls of the caudate as blue dots. b, c. Each EdU-labeled nucleus is shown as a colored dot with colors assigned according to the number of neighboring EdU-labeled nuclei located closer than 200 µm to this nucleus. Scale is shown on the right. EdU-labeled nuclei in the LatW and in the dorsal and lateral walls of the caudate are shown as they are located in the mouse brain (b) or virtually separated by 1 mm (c). B. Distribution of proliferating cells in the lateral walls of the lateral ventricles formed by the medial wall of the caudate and in the dorsal and lateral walls of the caudate. Proliferating cells in the medial caudate wall facing the lateral ventricles are shown with yellow arrows and in the dorsal and lateral walls with blue arrows. The point of transition between caudate walls facing the lateral ventricle and other walls is shown with the gray arrows. Sagittal sections. Section positions are shown on diagrams on the top. The red arrow on panel d shows the point of the RMS departure from the CMS. Lateral Ventricles (LV), Caudoputamen (CP), Rostral Migratory Stream (RMS). C. Distribution of proliferating cells in the LatW and in the dorsal and lateral walls of the caudate (Caud) along the anteroposterior axis of the brain. The number of EdU-labeled nuclei in a 50 µm brain section is shown along the vertical axis of the charts. The distance from the anterior end of the brain is shown along the horizontal axis of the charts. D. Density of proliferating cells in the LatW and Caud along the anteroposterior axis of the brain. The average number of neighboring EdU-labeled nuclei located closer than 200 µm to each EdU-labeled nucleus in a 50 µm brain section is shown along the vertical axis of the charts. The distance from the anterior end of the brain is shown along the horizontal axis of the charts. E–G. Distribution of proliferating cells in walls of the lateral ventricles. E, F. Distribution of proliferating cells in the medial wall of the lateral ventricles (MedW) and at the place of the lateral and medial wall fusion in the anterior part of the lateral ventricles (FusW) (E) and in the dorsal wall of the lateral ventricles (DorW) (F). The number of EdU-labeled nuclei in a 50 µm brain section is shown along the vertical axis of the charts. The distance from the anterior end of the brain is shown along the horizontal axis of the charts. G. Distribution of proliferating cells in walls of the lateral ventricles. (a–f. Lateral view; g. Frontal view and h. Dorsal view). The cell location is indicated on the figure. Each EdU-labeled nucleus is shown as a colored dot with colors assigned according to Table 2. Abbreviated names are given according to Table 2. 120 days-old mouse one hour post EdU injection.

**Figure 10:**
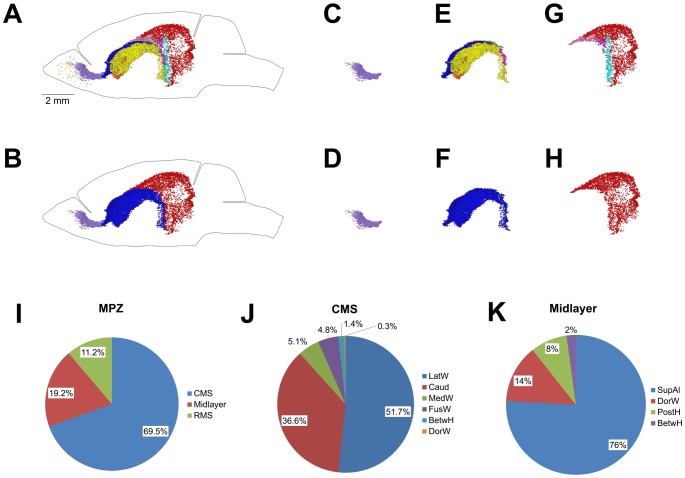
Distribution of proliferating cells in the Main Proliferative Zone (MPZ). A–H. Distribution of proliferating cells in the MPZ (A, B), RMS (C, D), CMS (E, F), and midlayer (G, H). Lateral view. Each EdU-labeled nucleus is shown as a colored dot with colors assigned according to Table 2 (A, C, E, G). Each EdU-labeled nucleus located in the RMS is shown as a lilac dot, in the CMS as a blue dot, and in the midlayer as a red dot (B, D, F, H). I–K. Pie chart showing the percentage of EdU-labeled nuclei located in different parts of the MPZ (I), CMS (J), and midlayer (K). Abbreviated names are given according to Table 2. 120 days-old mouse one hour post EdU injection.

**Figure 11:**
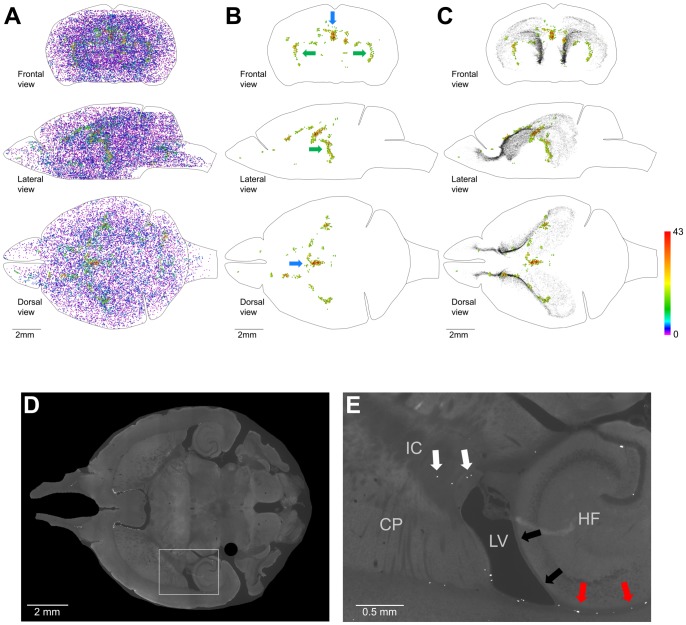
Distribution of proliferating cells outside of the Main Proliferative Zone (MPZ) and the dentate gyrus of the hippocampus (DG). A–C. Volume number density of proliferating cells. Each EdU-labeled nucleus is shown as a colored dot with colors assigned according to the number of neighboring EdU-labeled nuclei located closer than 200 µm to this nucleus. Scale is shown on the right. A. All EdU-labeled nuclei located outside of the MPZ and DG. B, C. 600 EdU-labeled nuclei that have 16 or more neighboring EdU-labeled nuclei located closer than 200 µm. EdU-labeled nuclei in the MPZ and DG are shown as black dots of smaller size (C). The brain shape is outlined with a black line. Frontal, lateral, and dorsal views of the brain are shown. D, E. Proliferating cells in the inner capsule are shown with white arrows (E). The presence of proliferating cells in places where the alveus is in contact with a layer of white matter formed by bundles connecting the thalamus with the cortex is shown with red arrows and the absence in places where no such contact with black arrows (E). The position of the images shown on panel E is indicated on panel D with the white rectangle. Caudoputamen (CP), Hippocampal Formation (HF), Lateral Ventricles (LV), Inner Capsule (IC). 120 days-old mouse one hour post EdU injection.

### Caudate Migratory Stream (CMS)

The majority of proliferating cells in the middle part of the MPZ are located in the LatWs and dorsal and lateral walls of the caudate (Caud). The cells in the LatW and Caud are located alongside each other (Figure 9A). We have not noticed any apparent changes in distribution or density of proliferating cells within the transition from the LatW into Caud (Figure 9B–D). In whole, proliferating cells in both structures appear to form one homogenous field of proliferating cells (Figure 9A). The lateral wall of the lateral ventricles is formed by the medial wall of the caudate (Figure 3B). Thus, proliferating cells in the LatW and Caud are located in walls of the caudate.

The lateral and medial walls in the anterior part of the lateral ventricles are often fused with each other (FusW) (Figure 1C). Proliferating cells located here are also located in the wall of the caudate (Figure 3B). Similarly, the majority of proliferating cells located between the posterior horn and the main body of the lateral ventricle (BetwH) are also located in the medial wall of the caudate (Figure 3C). Thus, the proliferating cells in the LatW, Caud, FusW and in the anterior part of the BetwH are all located in the walls of the caudate.

Proliferating cells are sparsely distributed in the medial walls of the lateral ventricle (MedW) with the exception of the most anterior parts (Figure 9E, G). The significant increase in the density of proliferating cells in the MedWs coincides with the fusion of the medial and lateral walls of the lateral ventricles and extends anteriorly from the fusion points (Figure 9E, G). Thus, the cells in the MedWs appear to be a spillover of proliferating cells moving along the LatWs into the olfactory bulbs. Proliferating cells in the dorsal wall of the lateral ventricles (DorW) form two areas of higher density. One of the areas is located at the very anterior end of the wall and another, more prominent, in the posterior half of the wall (Figure 9F, G). The anterior area appears to be the place where cells moving along the lateral and medial walls are meeting together at the anterior end of the lateral ventricle (Figure 9G).

The cells in these six structures occupy the middle part of the MPZ (Figure 10E, F). About 95% of them are located in the walls of the caudate, and the remaining 5% located in the dorsal and medial walls of the lateral ventricles appear to be a spillover from the medial walls of the caudate. The cells have a low density and number at the posterior end of the caudate that gradually increase by about ten times toward the anterior end of the caudate (Figure 7C, I, J). Such a cell distribution resembles a river that is narrow and shallow at the source and becomes larger as it progresses. Taking into account that practically all these cells are located in the walls of the caudate we named this part of the MPZ as the caudate migratory stream (CMS) (Figure 10E, F, J).

### Midlayer

The posterior part of MPZ has a significantly lower density of proliferating cells compared to that in the RMS and CMS (Figure 7A–E). About three quarters of these cells are supra alveus cells (supAl) (Figure 10K). These cells are located in a very narrow layer of tightly packed cells located on the surface of the alveus (Figure 3E, F). Proliferating cells are not present on the whole surface of the alveus. They are present only in places where the alveus is in contact with a layer of white matter formed by bundles connecting the thalamus with cortex (Figure 11E).

The SupAl field of proliferating cells borders fields of proliferating cells located in the posterior part of the DorWs and in the posterior horn of the lateral ventricles (PostH) (Figure1C; 10G). The posterior part of the DorW and the lateral wall of the horn are composed of the layer of white matter formed by the bundles connecting the thalamus with cortex. In additional, a small fraction of the cells in the BetwH that are not located in the CMS are also located on the inner surface of these bundles. Thus, proliferating cells in the posterior part of the MPZ are located on the inner surface of the white matter layer formed by thalamo-cortical fibers. Because these cells principally form a thin layer of proliferating cells located between two layers of white matter we name this part of the MPZ as the midlayer.

Volume density of proliferating cells in the midlayer is low compare to other parts of the MPZ with the exception of the cells at the anterior edge of the DorWs (Figure 7D, K). This is due to the proximity of these cells to the CMS which has a much higher density of proliferating cells. If we remove 250 cells that are close to the CMS it becomes apparent that the density of proliferating cells in the midlayer remains fairly constant along the antero-posterior axis (Figure 7K, L). Because the midlayer is very thin we can calculate that the areal density of proliferating cells in the midlayer is about 300 cells per square millimeter. That is one and a half times higher than observed in the DG (190 cells/mm2).

### Other Proliferating cells in the adult mouse brain

Proliferating cells outside of the aggregation are distributed throughout all parts of the adult mouse brain with a volume density much lower than in the aggregation (Figure 4C; 7E). The proliferating cells in the cortex are distributed with very low density (Figure 11A). However, some areas inside the brain appear to have a higher density of proliferating cells (Figure 11A). We selected 600 cells with the highest number of neighboring cells (from 43 to 16 neighbors) and visualized their distribution (Figure 11B). The most prominent aggregation of these proliferating cells is located in the choroid plexus in the third ventricle (Figure 3A). Another prominent aggregation is located in the temporal area of the brain where proliferating cells form arcs (Figure 11B). The cells in this aggregation are located in the inner capsule near the bottom of the lateral ventricles (Figure 11B, D, E). Both of these areas appear not to be connected with the aggregation (Figure 11C).

## Discussion

Adult neurogenesis is a complex process. We can divide it into three distinct stages. The first stage is the production of neural precursors. The second stage is the movement of neural precursors from their place of birth toward the site where they will be incorporated into the adult mouse brain. The third stage is the differentiation of neural precursors into particular types of neurons and incorporation into existing neural circuits. We focused on the first stage in our study. Neural precursors in the adult mouse brain are produced by division of astrocyte-like cells and at birth they continue to express glial markers [4]–[6]. Only several days later with the appearance of doublecortin expression do they start to clearly manifest differentiation toward the neural phenotype [12]–[14]. The presence of doublecortin expressing cells in all parts of the MPZ shows that at least some of proliferating cells in each part of the MPZ will differentiate toward the neural phenotype.

We found that proliferating cells are distributed throughout the entire adult mouse brain (Figure 2B). About two thirds of these cells are located in the aggregation that spans numerous locations in the brain (Figure 4B). Analysis of the aggregation shows that it is composed from two continuums of proliferating cells. The smaller one contains about 5% of the proliferating cells in the aggregation and corresponds to the subgranular zone (SGZ) of the DG (Figure 4D, F). The larger one, the MPZ, contains the remaining 95% of the proliferating cells (Figure 4E, F). The MPZ can be divided into the RMS, CMS and midlayer. The CMS occupies the middle part of the MPZ. The stream of proliferating cells emanating from the most anterior part of the caudate toward the OB forms the RMS. The thin layer of proliferating cells extending posteriorly from the CMS forms the midlayer (Figure 10). We have not found any additional aggregations of proliferating cells that could suggest the existence of other major neurogenic zones in the adult mouse brain.

From the discovery of adult neurogenesis, the presence of proliferating cells in the LatWs becomes apparent. The LatWs are clearly identifiable on the sections of the brain and the abundance of proliferating cells within them is obvious. These findings led to models of adult neurogenesis depicting the LatWs as the principal place for production of new neurons in the adult mouse brain [5], [6]. In contrast, we found that only about one third of proliferating cells in the MPZ are located in the LatWs. The distribution of proliferating cells in the LatWs is indistinguishable from the distribution of proliferating cells in the caudate walls not facing the ventricle (Figure 9A, B). The cell density increases in the LatW and Caud toward the anterior end at a very similar rate and extent (Figure 9D). Average cell density is even higher in the caudate walls that are not facing the ventricle (Caud, FusW) compare to that in the LatW (Figure 7F). These data show that proximity to the wall of the lateral ventricles has no apparent effect on proliferating cells in the caudate walls. Thus, the cells in the LatWs appear to be an integral part of the CMS because they do not have any distinguishing properties that could warrant viewing them as a unique part of the MPZ or even CMS.

Proliferating cells in the LatWs of the adult mouse brain produce neural precursors that migrate into the OB where they differentiate into interneurons (reviewed in [5], [6]). During mouse brain development, precursors for olfactory interneurons are produced in the lateral ganglionic eminences (LGE) [15], [16]. The LGEs together with medial and caudal ganglionic eminences appear as a bulge of proliferating cells located in the dorsal wall of the developing caudate (as seen in Fig.1 of Rudolph et al., 2010 [17] or in Andrews et al., 2007 [18]). The location in the caudate wall is even more apparent in the developing human brain (as seen in Fig.1 in Del Bigio, 2011 [19] or Kostovic and Vasung, 2009 [20], or in Fig. 5 of Ohira et al., 2002 [21]). As the brain continues to develop, the caudate expands and together with the putamen forms lateral walls of the lateral ventricle where production of precursors for the OB interneurons continues. It also continues in the dorsal and lateral walls of the caudate that have no direct exposure to the ventricles (Figure 9A, B). Therefore, it appears that the walls of the caudate maintain a neurogenic niche for production of olfactory interneurons during brain development and in the adult mouse brain.

The presence of proliferating cells in the dorsal part of the midlayer was originally reported by Nakatomi and colleagues (2002) [22]. Later, Seri and colleagues (2006) [23] showed that proliferating cells are also present in the ventral and posterior part of the midlayer. They named this proliferative area the subcallosal zone (SCZ). We find this name confusing for several reasons. First, the subcallosal term was used in Gray's Anatomy of the Human Body about a hundred years ago and later it was incorporated into Terminologia Anatomica to describe a small area below the anterior end of the corpus callosum. There is a substantial body of research devoted to the studies of this subcallosal area. Second, proliferating cells are located below the layer of white mater bundles connecting the thalamus with cortex. This can be clearly observed in the dorsal wall of the lateral ventricles. The middle part of the dorsal wall is formed by the corpus callosum, and this part of the wall is devoid of proliferating and DCX expressing cells (Figure 6D, Figure 9F, G). The posterior part is formed by thalamo-corticle bundles and has an abundance of proliferating and DCX expressing cells (Figure 6C, Figure 9G). An example of the distribution of this type of bundlescan be seen in the Mouse Brain Connectivity Atlas (experiment 292320572-LGd, Allen Institute for Brain Science, http://www.alleninstitute.org/). Third, because the proliferating cells are confined to a thin layer (Figure 3E, F), that area should not be referred to as a zone as this suggests an area of considerable thickness and some 3D organization. We propose to use the term “midlayer” for this part of the MPZ since the proliferating cells are located between two layers of white matter bundles. This is a new term, and its use should not create any confusion with the classical use of the "subcallosal zone/area".

Distribution of proliferating cells in the midlayer differs from other parts of the MPZ. The density of proliferating cells in the midlayer is significantly lower than in the CMS and RMS (Figure 7E). However, it is still higher than in the DG (Figure 7E). In addition, this density is maintained fairly evenly along the antero-posterior axis. This is in a sharp contrast to significant changes in cell density in the CMS and RMS (Figure 7G, I, K). The presence of the midlayer is not unique to the adult mouse brain, and in fact appears to be a standard structure of the adult mammalian brain. The midlayer-like structures are also present in the adult brain of primates [24], bats [25], rabbits [26], and rats [27], [28]. While the midlayer contains four times more proliferating cells than the DG (Table 2), it appears that the majority of neural precursors produced in the midlayer undergo programmed cell death [29] and its contribution to adult neurogenesis may be limited.

Aggregations of proliferating cells were reported in the walls of the third ventricle on the ventral side of the brain [30], [31] and hypothalamus parenchyma [32]. These proliferative zones are supposed to be producing precursors for new hypothalamic neurons [33]. We have not observed any prominent accumulation of proliferating cells at these two locations. Our data support the observation that proliferative activity in these zones decreases rapidly in mice after birth and becomes practically undetectable in mice at the age of 45 days [31]. Thus, active hypothalamic neurogenesis may be limited to a couple of months after birth.

We found that 16,042 proliferating cells are located throughout all parts of the mouse brain outside of two neurogenic areas. The majority of these cells are likely to be oligodendrocyte precursors (OPCs) [34] that produce oligodendrocytes and a small number of neurons [35]. The remaining cells are likely to be self-renewing microglial cells [36], endothelial cells and pericytes. We noticed two areas in the inner part of the brain that appear to have a higher density of proliferating cells. One of them is the choroid plexus in the third ventricle (Figure 3A; Figure 11B) which indicates that the choroid plexus is undergoing continuous renewal in the adult brain. The other area is the inner capsule (Figure 11B, D, E) which is formed by white matter and these proliferating cells are most likely OPCs. On the whole the presence of proliferating cells in all parts of the brain outside of two neurogenic areas shows that cell replacement continues in the entire adult mouse brain. However, we still do not know how many and what types of neurons if any are among these cells.

Adult neurogenesis in the healthy brain is limited to the production of new olfactory interneurons and hippocampal granule cells. There is also a possibility of production of a small number of new interneurons in the cortex and other parts of the brain [8], [37]. However, there is a hope that variety and number of neurons produced in the adult brain can be expanded and adult-born neurons will be used in humans to repair brain after trauma, stroke and damage inflicted by neurodegenerative diseases. We found that about 30,000 EdU-labeled cells are located in the MPZ (Table 2). EdU is labeling proliferating cells during the S phase of cells cycle by incorporating into replicating chromosomal DNA. The length of the S phase in cells located in the lateral walls of lateral ventricles is measured to be approximately four hours [38]. Thus, we can estimate that around 180,000 new cells are produced daily in the MPZ in the brain of 120 day-old male mouse. The mouse neocortex is built from approximately ten million neurons [39]. Therefore, production of new cells in the MPZ, even without additional stimulation, should be sufficient to replace all neurons in the neocortex within two months. However, not all proliferating cells in the MPZ will differentiate into neurons and many of new neurons under normal circumstances will die before they will be fully incorporated into brain circuitry [40]. And even if these obstacles are solved, we still need to develop techniques to direct movement of all these cells to the proper regions in the neocortex, to ensure that they are differentiating into the correct type of neurons and are reestablishing the connections of the neurons in the cortex they replace. It is important to note, however that in humans, the possible contribution of adult neurogenesis for brain repair may be different than that in mice.

## Methods

### Ethics Statement

All experiments with mice were conducted following the NIH and international guidelines and with veterinarian supervision. All experimental procedures were approved by The Institutional Animal Care and Use Committee (Department of Veterans Affairs, ENRM VA Hospital IACUC, Protocol SE-08-13-96).

C57BL/6J male mice were injected intraperitoneally with 50 mg/kg EdU (Invitrogen). Mice were euthanized at time points described in the article and transcardially perfused first with 20 ml of cold PBS with 10 U/ml of heparin and then with 120 ml of cold PBS with 4% formaldehyde. Brains were extracted and incubated in PBS with 4% formaldehyde at +4C for a day and then transferred into 100 mM phosphate buffer, pH 7.4, with 20% of glycerol and 2% of DMSO for at least two days before cutting. Frozen 50 µm serial brain sections were cut in the transverse plane with a sledge microtome. About 154 sections were produced from each studied mouse brain. Transverse sections were used because they maintain their shape well, which simplifies image registration. Brains were cut into 50 µm sections because sections of this thickness are easily handled during the staining and mounting processes while still allowing complete staining through the entire section. EdU staining was performed using a Clik-iT EdU imaging kit with Alexa-Fluor 555 (Invitrogen) following the manufacturer's instructions. For doublecortin (Cell Signaling #4604) and phospho-histone H3 (Ser10) (Cell Signaling #9716) staining we used antibodies diluted at 1∶1000. For Hoechst staining, sections were incubated for one hour in PBS with 0.8 µg/ml Hoechst 33342 and 0.5% Triton X100.

To visualize the distribution of proliferating cells in the entire mouse brain, we cut the entire brain transversely in 50 µm sections and stained all sections for EdU. Stained sections were mounted on 24 microscope slides (25×75 mm), and each slide was scanned using the Zeiss AxioImagerZ2 microscope with a 5X objective. We used a 5X objective for image acquisition because its focal depth exceeds the thickness of brain sections and the spatial resolution is sufficient for unambiguous detection of EdU-labeled nuclei. Each scan produced a 16-bit composite image of the entire microscope slide consisting of 600 individual images. Composite images were stitched using the Grid Stitching Plug-in [41] for the Fiji image processing package. Images for individual sections were cut out from stitched images and ordered according to the position in the brain using the Fiji image processing package. Then, the images were registered manually using Adobe Photoshop. Coordinates of EdU-labeled nuclei on each section were obtained by using the Find Maxima Process with the tolerance parameter set to 1500 (Fiji image processing package) and combined into a single point cloud file (apo) using Microsoft Excel. The tolerance parameter was set to 1500 because this not only allowed us to automatically identify about 90% of EdU-labeled nuclei but at the same time also produced a low rate of false positive identifications that we manually removed. To visualize point clouds of EdU-labeled nuclei, we used the Vaa3D visualization program [42]. We defined the number and reconstructed the distribution of proliferating cells in four mouse brains. One of these mice was 60 day-old (60D mouse), two were 120 day-old (120D-A and 120D-B mouse), and one was 240 day-old (240D mouse). Classification of proliferating cells and analysis of their distribution was performed for the 120D-A mouse brain.

Microsoft Excel was used for all data analysis and chart drawing. Adobe Photoshop and Microsoft PowerPoint were used for figure preparation. All microscopic images shown in the figures, except those shown in Figure 1B and 1D, are obtained using a 5X microscopic objective. Microscopic images shown in Figure 1B are obtained using a 40X microscopic objective. Maximum intensity projections of the brain section stained for EdU shown in Figure 1B were obtained using a 40X objective with Apotom2 and ZEN image analysis software (Zeiss).
